# Successes and Challenges of Sustaining Community-Based Programs for People Who Live Alone with Dementia

**DOI:** 10.1093/geroni/igaf122.413

**Published:** 2025-12-31

**Authors:** Kate Gordon, Yoon Chung Kim, Lirisha Tuladhar, Heather Menne, Michael Lepore

**Affiliations:** Splaine Consulting, Columbia, Maryland, United States; University of Maryland School of Medicine, Baltimore, Maryland, United States; Miami University, Oxford, Ohio, United States; Miami University, Oxford, Ohio, United States; University of Massachusetts Amherst, Amherst, Massachusetts, United States

## Abstract

Approximately a quarter of older adults with dementia or cognitive impairment live alone in the US. To address the needs of people living alone with dementia (PLAWD), the Administration for Community Living requires PLAWD-focused services in its Alzheimer’s Disease Program Initiative (ADPI) to address service gaps and improve care delivery for PLAWD. We examined 59 ADPI grants to determine the types of services delivered, and successes and challenges faced in service delivery. Grantees provided care management and/or delivered services such as companionship and support for socialization through buddy programs, phone calls, social and wellness programming, and regular home visits. This study explores the post-grant period experiences of community-based organizations that received federal funding. The study team conducted semi-structured, online interviews with 13 of the 59 grant program directors representing multiple regions of the US. Interviews were audio/video-recorded, transcribed, and manually coded by team members using an inductive approach to identify key themes. Findings highlight the factors that impacted the long-term sustainability of PLAWD-focused services after federal funding ceased. Grantees implemented strategies to maintain and expand services including modifying services, partnering with local service providers and leveraging existing relationships. Findings demonstrate that a variety of services have been successfully sustained for PLAWD. However, common challenges are experienced in sustaining service delivery after initial grant funding ends. PLAWD often lack local care partners and need specialized services and support, including care management, care monitoring, and socialization. Different approaches used and challenges faced in sustaining grant-initiated community programs for PLAWD are discussed.

